# Electro-Acupuncture Alleviates Cisplatin-Induced Anorexia in Rats by Modulating Ghrelin and Monoamine Neurotransmitters

**DOI:** 10.3390/biom9100624

**Published:** 2019-10-18

**Authors:** Ji Yun Baek, Tuy An Trinh, Wonsang Huh, Ji Hoon Song, Hyun Young Kim, Juhee Lim, Jinhee Kim, Hyun Jin Choi, Tae-Hun Kim, Ki Sung Kang

**Affiliations:** 1 College of Korean Medicine, Gachon University, Seongnam 13120, Korea; wldbsttn@naver.com (J.Y.B.); tuyan2308@gc.gachon.ac.kr (T.A.T.); wshuh@gc.gachon.ac.kr (W.H.); jhsong.john@gmail.com (J.H.S.); 2 Department of Food Science, Gyeongnam National University of Science and Technology, Jinju 52725, Korea; hykim@gntech.ac.kr; 3 College of Pharmacy and Institute of Pharmaceutical Sciences, CHA University, Seongnam 13488, Korea; juheelim@cha.ac.kr (J.L.); kjh05267@naver.com (J.K.); hjchoi3@cha.ac.kr (H.J.C.); 4 Korean Medicine Clinical Trial Center, Korean Medicine Hospital, Kyung Hee University, Seoul 02447, Korea

**Keywords:** electro-acupuncture, 5-hydroxytryptamine, ghrelin, neuropeptide Y, pro-opiomelanocortin, cisplatin, anorexia, cancer

## Abstract

Anorexia is common in patients with cancer, mostly as a side effect of chemotherapy. The effect of electro-acupuncture (EA) on ameliorating cancer-related symptoms have been studied in animal models and in clinical trials. The aim of this study was to determine optimal conditions for the application of EA to alleviate anorexia, followed by the study of molecular mechanisms affecting its therapeutics. Anorexia was induced in male Wistar rats by injecting cisplatin, which was then followed by EA treatment at CV12, the acupuncture point located in the center of the abdominal midline. Body weight and food intake were measured daily throughout the duration of the study. The levels of monoamine neurotransmitters in the plasma were quantitatively analyzed by HPLC-ECD. Gastrointestinal hormone concentrations were elucidated with ELISA kits. RT-qPCR was performed to evaluate the mRNA expression of ghrelin (GHRL), neuropeptide Y (NPY), and pro-opiomelanocortin. The expression of c-Fos in the nucleus tractus solitarii was detected using western blotting analysis. The optimal conditions of EA to alleviate anorexia in rats was determined to be 1 unit for intensity and 10 Hz for frequency. EA treatment at CV12 reduced the levels of plasma monoamine neurotransmitters 5-hydroxytryptamine, 5-hydroxyindoleacetic acid, dopamine, and norepinephrine; as well as stimulated the expression of GHRL and NPY to alleviate cisplatin-induced anorexia in rats. EA stimulation at CV12 could be used to treat cisplatin-induced anorexia in rats.

## 1. Introduction

Cancer is a major cause of death in Korea, accounting for 27.8% of total mortality in 2016 [1]. The forecast for newly diagnosed cases of cancer in Korea for 2019 is 221,347 [2]. Besides cancer developing to advanced stages, malnutrition of patients also contributes to mortality [3]. Anorexia is commonly seen in patients with cancer, especially those undergoing chemotherapy. Anorexia is defined as the loss of interest in food, expressed through loss of appetite, early satiety, or alterations in the taste and odor of food [4]. Anorexia is normally accompanied by cachexia, which causes weight loss, mainly from adipose tissues and skeletal muscles [3]. Approximately 65% of terminally ill patients with cancer suffer from anorexia [5]. It leads to weakness of the body and affects the recovery capacity, as well as the survival rate of those affected.

Anorexia is associated with the development of cancer, like a physiological response to tumor growth. Aside from anorexia, nausea and vomiting are also common side effects of chemotherapy and radiotherapy [6]. Multiple factors contribute to the pathogenesis of anorexia in cancer through hypothalamic neuronal signaling pathways such as gastrointestinal hormones, energy signals, neurotransmitters, neuropeptides, and hypothalamic neuroimmune interactions [5,6]. Therapeutic strategies to treat anorexia related to cancer focus on improving appetite and neutralizing metabolic disorders. The common drugs used to stimulate appetite are progestins, cannabinoids, cyproheptadine, corticosteroids, GHRL, and melanocortin antagonists [5,6,7].

Oriental medicine is commonly used as an adjunctive therapy for the treatment of cancer. Along with herbal medicine, acupuncture may be used to alleviate the side effects of anticancer therapies. Acupuncture has been practiced in Asian countries for thousands of years. More recently, various methods of acupuncture have been developed such as manual acupuncture, EA, transcutaneous electric nerve stimulation, acupressure, auricular acupuncture, and moxibustion. Clinical trials have suggested that EA was able to reduce acute vomiting caused by chemotherapy [8,9], improve insomnia [10], and relieve pancreatic pain caused by cancer [11].

The effect of the underlying mechanisms of EA on ameliorating cancer-related symptoms has been studied in animal models. EA at CV12 alleviated cisplatin-induced nausea in rats by reducing the 5-HT level in the duodenum along with inhibiting the expression of c-Fos in the NTS [12]. EA at ST36 decreased the gastrointestinal hormone levels of GHRL, peptide YY, and glucagon-like peptide-1, which contribute to the ameliorating effect on cisplatin-induced dyspepsia in rats [13]. Previously, the therapeutic effects of EA on cisplatin-induced anorexia in rats were evaluated using various acupuncture points such as CV12, PC6, and ST36 [14]. This study aims to ascertain the optimal conditions for EA treatment to alleviate anorexia using evidence from molecular mechanism studies for support.

## 2. Materials and Methods

### 2.1. Animals

Male Wistar rats (body weight, 200–240 g; age, 7 weeks old) were procured from Orient Bio Co., Ltd. (Seongnam, Korea) and used in this study.

All procedures involving the use of live animals described in the present study were approved by the Institutional Animal Care and Use Committee of Gachon University (Seongnam, Korea; approval no. GIACUC-R2015011) in December 2015. The rats were housed in normal conditions and were exposed to a 12 h light/dark cycle at 23 ± 2 °C and 55 ± 5% RH. The rats were fed a standard chow diet containing 10% fat ad libitum for one week before the experiments were performed.

### 2.2. Description of Study

This study included two experiments, one to evaluate the effects of different EA conditions on cisplatin-induced anorexia, and another to study the pharmacological mechanisms of the optimal EA conditions obtained from the result of the first experiment. In the first experiment, two tests were designed to ascertain for the optimal intensity and frequency of EA conditions. For the intensity study, the rats were randomly divided into 3 groups (*n* = 3): Cisplatin (injected with cisplatin, without EA), Cisplatin + CV12 (1 unit) (injected with cisplatin, EA at the intensity of 1 unit), and Cisplatin + CV12 (4 units) (injected with cisplatin, EA at the intensity of 4 units). Similarly, the rats were randomly divided into 3 groups (*n* = 3) for the frequency study as well: Cisplatin (injected with cisplatin, without EA), Cisplatin + CV12 (10 Hz) (injected with cisplatin, EA at the frequency of 10 Hz), and Cisplatin + CV12 (100 Hz) (injected with cisplatin, EA at the frequency of 100 Hz). The effect of each EA condition was assessed through the change in body weight and food intake.

Based on the results obtained from the first experiment, EA at frequency 10 Hz and intensity 1 unit was chosen for the second part of the experiment. The rats were grouped into 3 (*n* = 3): Vehicle (injected with saline; EA intensity 1 unit, frequency 10 Hz at the non-acupoint), Cisplatin (injected with cisplatin; without EA), and Cisplatin + CV12 (injected with cisplatin; EA intensity 1 unit, frequency 10 Hz at the CV12 acupoint). After 3 days, the rats were sacrificed and the blood, duodenum, hypothalamus, and brain stems were collected for further analysis. The details of the experimental design are mentioned in Table 1.

### 2.3. Anorexia Model

The rats were allowed to adapt to laboratory conditions for one week prior to the experiment. Anorexia was induced via a single intraperitoneal (i.p.) injection of 6 mg/kg cisplatin (saline was injected in the control group).

### 2.4. Measurement of Body Weight and Daily Food Intake

One day before the administration of cisplatin, the rats were placed individually in acrylic metabolic cages (JD-C-66; Jeung-Do Bio and Plant Co., Ltd., Seoul, Korea) until the end of the experiment. The body weight, food intake, water intake, and urine output were measured every 24 h. Food intake was calculated as the difference between the food provided initially and the unconsumed food.

### 2.5. Electro-acupuncture Treatment

The CV12 acupuncture point was selected and located according to the acupuncture atlas for rats [15]. Disposable stainless-steel acupuncture needles (0.25 × 40 mm; Dongbang Acupuncture Inc., Seoul, Korea) were inserted to a depth of 2 mm in the center of the abdominal midline and 1 cm below. The acupuncture point was stimulated using an EA system (ES-160; ITO Physiotherapy and Rehabilitation Co., Ltd., Tokyo, Japan) for 10 min at the indicated intensity and frequency. The peak amplitude applied was 16 mA for the low intensity stimulation and 32 mA the for high intensity stimulation. The frequency applied for the low and high stimulation were 10 Hz and 100 Hz respectively. During the EA process, the rats were anesthetized with 2.8% isoflurane (Hana Pharm Co., Ltd., Hwaseong, Korea), using oxygen as a carrier gas, at a flow rate of 400 mL/min. Inhalational anesthetics were provided mechanically (Parkland Scientific Inc., Coral Springs, FL, USA).

### 2.6. Quantitative Analysis of Monoamine Neurotransmitters in Plasma

Blood samples were collected from the heart of the anesthetized rats into a tube containing EDTA anticoagulant. After centrifugation (1500× *g*, 4 °C for 10 min), 3.6 μL of 70% perchloric acid was added to 100 μL of the plasma supernatant. The mixture was centrifuged (3000× *g*, 4 °C for 10 min) and filtered through a 0.22 μm membrane filter. Further, 20 μL of the filtrate was injected into a Nova-Pak C18 column (60 Å, 4 μm, 3.9 mm × 150 mm; Waters Corp., Milford, MA, USA) and analyzed using HPLC [16]. The flow rate of the mobile phase (85 mM citrate, 100 mM sodium acetate, 0.9 mM sodium octyl sulfate, 0.2 mM EDTA, and 12% methanol, adjusted to pH 3.7) was 1.0 mL/min. Monoamine neurotransmitters serotonin (5-hydroxytryptamine, 5-HT), 5-hydroxyindoleacetic acid (5-HIAA), dopamine (DA), and norepinephrine (NE) were detected and quantified by ECD (2465; Waters Corp., Milford, MA, USA).

### 2.7. Measurement of Gastrointestinal Hormone Levels in the Plasma

Blood samples were mixed with EDTA after collection to prevent coagulation. They were centrifuged immediately (1500× *g*, 4 °C for 10 min) to obtain a supernatant. The plasma samples were stored at −80 °C until required. The levels of GHRL and CCK were determined using ELISA kits (EK-069-04 and MM-402 respectively; Mitsubishi Kagaku Iatron Inc., Tokyo, Japan) according to the manufacturer’s instructions.

### 2.8. RT-qPCR

The duodenum was dissected to explore the mRNA expression of GHRL by RT-qPCR and the hypothalamus was harvested for the detection of NPY and POMC (pro-opiomelanocortin). The total RNA was extracted from tissues using the Tri-RNA reagent (Favorgen Biotech Corp., Kaohsiung, Taiwan) following which, cDNA was synthesized using a RevertAid First Strand cDNA Synthesis Kit (Thermo Fisher Scientific Inc., Waltham, MA, USA) according to the manufacturer’s protocols [17]. Reaction mixtures were prepared with cDNA and PowerUp SYBR Green Master Mix (Thermo Fisher Scientific Inc.) and a set of primers for the selected genes. The specific sequences of the primers are listed in Table 2. The qPCR was conducted using a QuantStudio 3 Real-Time PCR System (Thermo Fisher Scientific Inc.) applying the following conditions: initial denaturation at 95 °C for 10 min; 45 cycles of annealing at 95 °C for 3 s, and 59 °C for 30 s. The relative mRNA expression level for each gene was analyzed using the 2–ΔΔCq method. β-actin was used as a reference gene.

### 2.9. Western Blotting Analysis

The brain stem was dissected and the NTS cells isolated, to detect c-Fos expression using the western blot technique. Tissues were homogenized in a non-ionic detergent buffer (1% Nonidet-P40, 5% glycerol, 1 mM EDTA, 25 mM Tris-HCl, and 150 mM NaCl at pH 7.5) and supplemented with a protease inhibitor mixture (Roche Co., Basel, Switzerland) for 20 min at 4 °C. The lysates were centrifuged (21,130× *g* at 4 °C for 10 min) to obtain a protein supernatant. The protein concentration of each whole-cell extract was determined using the Bio-Rad Protein Assay Kit (Bio-Rad Laboratories Inc., Hercules, CA, USA). Equal amounts of protein from each protein extract (30 μg/lane) were separated by electrophoresis on an 8% sodium dodecyl sulfate-polyacrylamide gel and blotted onto PVDF transfer membranes. Epitope-specific primary antibodies including c-Fos and α-actin conjugated with secondary antibodies (Cell Signaling Technology Inc., Danvers, MA, USA) were used to label the target proteins. The bound antibodies were visualized with Immobilon Western Chemiluminescent HRP Substrate (EMD Millipore Co., Billerica, MA, USA) and analyzed using a digital imaging system (LAS-4000; GE Healthcare Bio-Sciences Corp., Little Chalfont, UK).

### 2.10. Statistical Analysis

A non-parametric method, the Kruskal–Wallis test, was first performed to analyze variance. The multiple comparisons test was performed by using Dunn’s pairwise test with Bonferroni adjustment as a post-hoc test. Further, the Mann–Whitney U test was used to compare the differences between experimental groups. All the statistical analyses were performed by SPSS Statistics 21.0 (IBM Corp., Armonk, NY, USA). A p-value less than 0.05 was considered statistically significant.

## 3. Results

### 3.1. Evaluation of the Optimal EA Condition to Alleviate Cisplatin-Induced Anorexia

**Figure 1:**
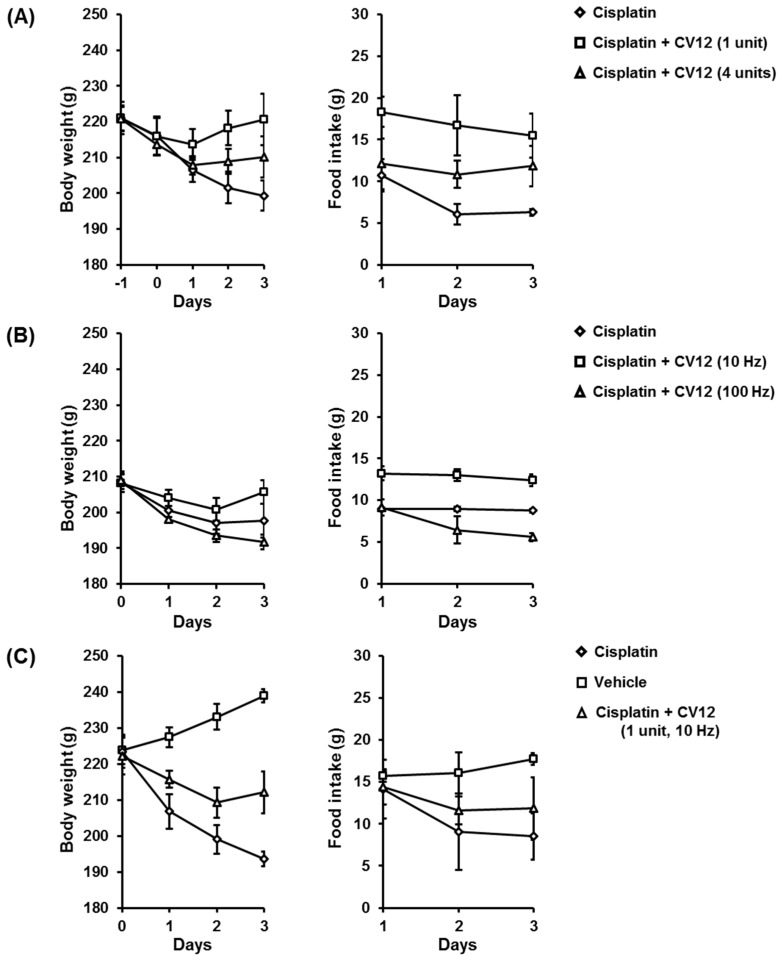
Changes in the body weight and food intake of rats during the experimental period. (**A**) Optimal intensity of EA, (*n* = 3): Cisplatin (injected with cisplatin, without EA), Cisplatin + CV12 (1 unit) (injected with cisplatin, EA at the intensity of 1 unit), and Cisplatin + CV12 (4 units) (injected with cisplatin, EA at the intensity of 4 units). (**B**) Optimal frequency of EA, (*n* = 3): Cisplatin (injected with cisplatin, without EA), Cisplatin + CV12 (10 Hz) (injected with cisplatin, EA at the frequency of 10 Hz), and Cisplatin + CV12 (100 Hz) (injected with cisplatin, EA at the frequency of 100 Hz). (**C**) Confirmation of the anti-anorexic effect of the chosen optimal conditions for EA (1 unit, 10 Hz), (*n* = 3): Vehicle (injected with saline, EA at the non-acupoint with the intensity of 1 unit and frequency of 10 Hz), Cisplatin (injected with cisplatin, without EA), and Cisplatin + CV12 (injected with cisplatin, EA at the CV12 acupoint with the intensity of 1 unit and frequency of 10 Hz). *p* < 0.05 was considered statistically significant.

The intensity and frequency of EA applied to stimulate acupuncture were modified and the therapeutic effect of each condition was assessed based on the changes in body weight and food intake between the experimental groups. The body weight of rats in all groups decreased one day after cisplatin was injected. In the test for the intensity of EA, the average weight was restored better in the group treated with EA at the intensity of 1 unit (Figure 1A) than the group treated with 4 units. The differences of average weight between day 0 and day 3 in each group were −16.72 g for Cisplatin, +4.73 g for Cisplatin + CV12 (1 unit), and −3.57 g for Cisplatin + CV12 (4 unit). Both EA groups also expressed improved nutritional tolerance after treatment with cisplatin.

The optimal frequency for EA was also studied. Stimulating EA at 10 Hz was seen to restore the body weight in the rats, whereas treatment at 100 Hz suppressed the recovery of the body weight even more than that observed in the control group (Figure 1B). The decrease in average weight between day 0 and day 3 was 10.62 g for Cisplatin, 2.52 g for Cisplatin + CV12 (10 Hz), and 17.13 g for Cisplatin + CV12 (100 Hz). Significant differences in food intake were not observed between the Cisplatin and the Cisplatin + CV12 (10 Hz) groups. In the Cisplatin + CV12 (100 Hz) group, the intake of food decreased.

Based on these results, the optimal condition for EA treatment was defined as 1 unit for intensity and 10 Hz for frequency. The anti-anorexic effect was reconfirmed using the same model. On treatment with EA (1 unit, 10 Hz), the body weight of the rats recovered better than the group treated only with cisplatin (Figure 1C). The reduction in average weight between day 0 and day 3 in each group was 29.83 g for Cisplatin and 10.05 g for Cisplatin + CV12 (1 unit, 10 Hz). EA treatment also improved food intake after the end of the experimental period.

### 3.2. Plasma Monoamine Neurotransmitters’ Concentrations

**Figure 2:**
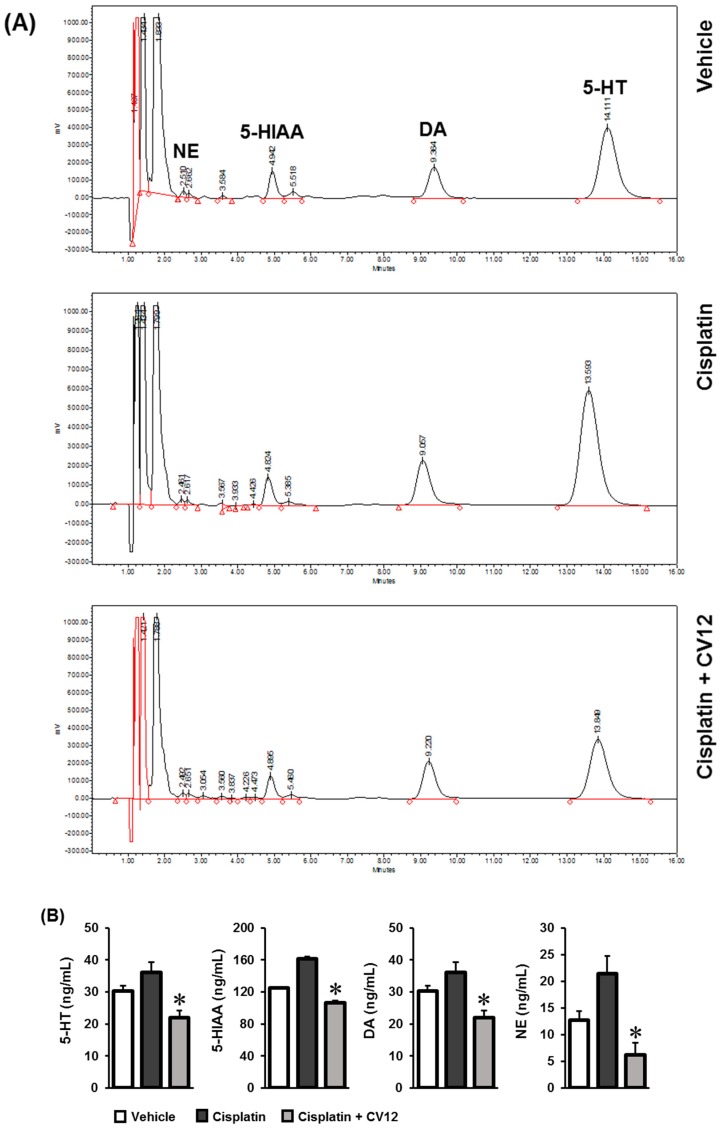
Quantitative analysis of monoamine neurotransmitters in plasma. The concentrations of 5-HT, 5-HIAA, dopamine (DA), and norepinephrine (NE) were quantitatively analyzed by an HPLC-ECD system. (**A**) The HPLC chromatograms. (**B**) The quantitative graphs. * p < 0.05 was considered statistically significant. (5-HT: 5-hydroxytryptamine; 5-HIAA: 5-hydroxyindoleacetic acid; DA: dopamine; NE: norepinephrine).

On treatment with cisplatin, the concentrations of 5-HT, 5-HIAA, DA, and NE in the plasma were significantly higher in the test group than the control group. EA stimulation at the CV12 acupoint reduced the levels of these plasma monoamine neurotransmitters, especially that of NE. The HPLC chromatograms and corresponding quantitative graphs of monoamine neurotransmitters are presented in Figure 2.

### 3.3. Gastrointestinal Hormone Levels in Plasma

**Figure 3:**
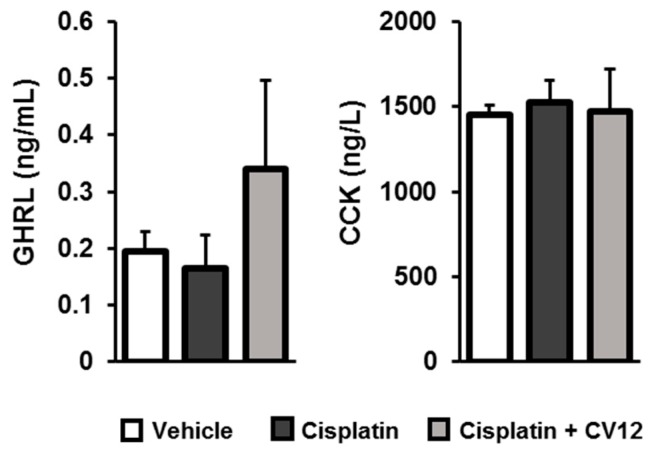
Gastrointestinal hormone levels in plasma. The levels of plasma GHRL and CCK were determined using ELISA kits. (GHRL: ghrelin; CCK: cholecystokinin).

The gastrointestinal hormone levels in plasma were measured using ELISA kits. The results are shown in Figure 3. The level of GHRL slightly increased in the EA-treated group compared with other groups. There were no statistical differences in the levels of CCK across the experimental groups.

### 3.4. mRNA Expression Levels of GHRL, NPY, and POMC

**Figure 4:**
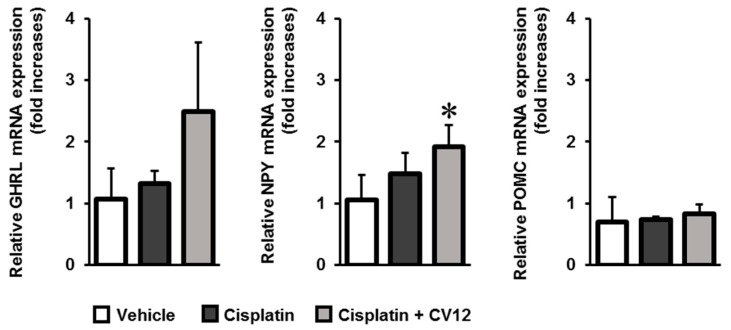
The mRNA expression levels of GHRL, NPY, and POMC were evaluated by RT-qPCR. * *p* < 0.05 was considered statistically significant. (GHRL: ghrelin; NPY: neuropeptide Y; POMC: pro-opiomelanocortin).

RT-qPCR was conducted to detect the mRNA expression levels of GHRL, NPY, and POMC. Upon treatment with EA at CV12, the expression of GHRL was stimulated in the duodenum. The expression of NPY from the hypothalamus slightly increased while that of POMC showed no change between experimental groups. The results of RT-qPCR are depicted in Figure 4.

### 3.5. c-Fos Expression in the NTS

**Figure 5:**
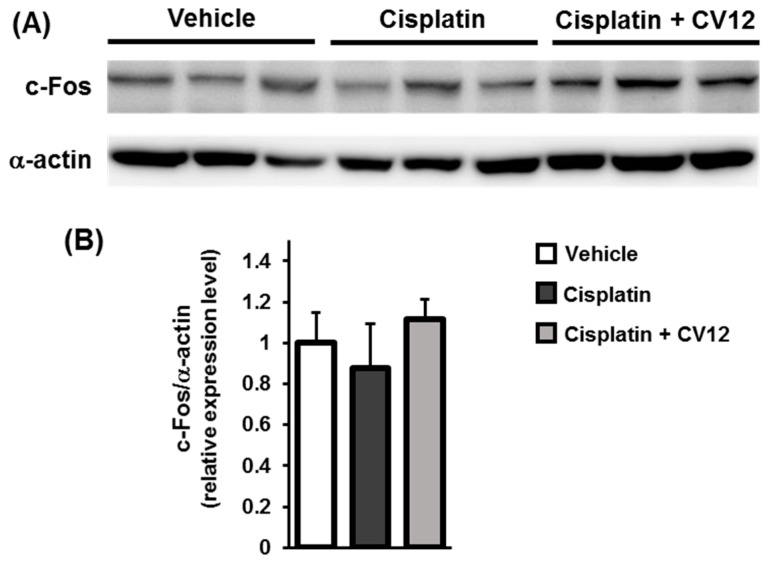
c-Fos expression in NTS cells. (**A**) Images of western blot analysis for the expression of c-Fos and α-actin. (**B**) The quantitative graph of c-Fos expression with α-actin as a loading control.

The protein expression of c-Fos in NTS cells was detected using western blot. The results showed no significant changes between experimental groups (Figure 5).

## 4. Discussion

Previous studies have compared the anti-anorexic effects of EA stimulation in different acupuncture points, including CV12, PC6, and ST36. Among these, CV12 was assessed as the most effective acupoint based on the food intake and recovery in body weight of the experimental models [14]. CV12, also known as *Zhongwan*, has been used in many research studies and clinical trials. EA at CV12 expressed an anti-emetic effect in rats by reducing the level of 5-HT in the duodenum and suppressing the expression of c-Fos in the NTS [12]. EA at ST25 and CV12 alleviated visceral pain in rats with post-inflammatory irritable bowel syndrome by inhibiting enterochromaffin cells and reducing the concentration of 5-HT [18]. Additionally, the therapeutic effect of acupuncture stimulation at CV12 for persistent hiccups [19] and pain in patients with cancer [20] was clinically studied. Hence the CV12 acupoint was chosen to study the optimal intensity and frequency for EA, followed by pharmacological mechanism studies. It was found that the optimal condition for EA stimulation to reduce cisplatin-induced anorexia in rats was 1 unit for the intensity and 10 Hz for the frequency. The effects on monoamine neurotransmitters, gastrointestinal hormones, and NTS cells were evaluated by molecular studies.

The monoamine neurotransmitters 5-HT, DA, NE, and epinephrine are involved in multiple functions such as the control of respiratory, cardiovascular, gastrointestinal, and psychomotor activities along with the regulation of hormone secretion, sleep, body temperature, and pain [21]. Serotonin, or 5-HT, affects satiety by activating multiple serotonergic receptors with discrete roles [22,23]. Studies performed in animal models have determined that 5-HT, at a high concentration, reduces total energy intake [24,25]. A major metabolite of 5-HT, 5-HIAA, has been used as an indicator for 5-HT activity in the central nervous system and the peripheral nervous system. The levels of 5-HT, 5-HIAA, DA, and NE in the plasma of experimental rats were quantified by HPLC-ECD analysis. Increased levels of 5-HT, 5-HIAA, DA and NE might exert metabolic effects contributing to the impairment characteristics for anorexia. The results obtained suggested that after cisplatin injection increased the concentrations of these plasma monoamine neurotransmitters, EA stimulation at CV12 was able to reduce them. This suggests that the therapeutic effect of EA at CV12 against cisplatin-induced anorexia in rats affects the signaling pathways of the serotonergic system.

Aside from monoamine neurotransmitters, gastrointestinal hormones and neuropeptides also participate in the regulation of appetite. GHRL is a key peptide in the gut that controls food intake and energy expenditure through its orexigenic effect. GHRL increases food intake in rats and stimulates the expression of NPY in the hypothalamus [26]. In contrast, CCK is the anorexigenic gastrointestinal hormone that causes a reduction in the meal size consumed and the duration of feeding [27]. Plasma GHRL levels were increased by EA treatment in the cisplatin-induced anorexic rats. The mRNA expression of GHRL in the duodenum tissue was also significantly stimulated after EA treatment. EA stimulation at CV12 did not cause any considerable difference in the concentration of plasma CCK between the experimental groups.

Hypothalamic neuropeptides act as key signaling agents in regulating appetite and feeding behaviors. NPY and orexin (also known as hypocretin) are orexigenic neuropeptides which stimulate feeding; whereas POMC, an α melanocyte-stimulating hormone, and corticotrophin-releasing hormone are considered anorexigenic neuropeptides [28]. The pathophysiological anorexic animal model illustrated that cisplatin reduced the gene expression of NPY, but enhanced the expression of POMC in the arcuate nucleus of the hypothalamus in rats [29]. EA stimulation increased the mRNA expression of NPY in the hypothalamus but did not affect the expression of POMC. The expression of c-Fos in NTS, a mediator of several neurotransmitter signaling pathways [30,31], was investigated but no significant changes occurred. Essentially, the anti-anorexic effect of EA treatment may cause the stimulation of GHRL which leads to up-regulation of NPY expression to improve appetite.

Our studies suggested that the body weight and appetite of rats significantly recovered by treatment with electro-acupuncture at CV12. The EA stimulation also induced changes in the levels of hormones and neurotransmitters related to appetite controlling. The acupuncture point CV12 has been studied in many clinical trials for the prevention of nausea and vomiting induced by chemotherapy [32,33,34,35]. In addition, the acupuncture treatment at CV12 showed a significant improvement in persistent hiccups [19] and pains [20] in patients with cancer.

## 5. Conclusions

Electro-acupuncture was used to improve the side effects of chemotherapy, including nausea, insomnia, cachexia, and pain, in patients with cancer. In this study, the optimal condition of EA to alleviate anorexia in rats was found to be 1 unit for intensity and 10 Hz for frequency. Pharmacological studies demonstrated that EA treatment at CV12 reduces the levels of plasma monoamine neurotransmitters 5-HT, 5-HIAA, DA, and NE; while simultaneously stimulating the expression of GHRL and NPY to alleviate cisplatin-induced anorexia in rats.

## Figures and Tables

**Table 1:** Evaluation of the effects of electro-acupuncture (EA) on a cisplatin-induced anorexic model.

Experimental Condition	Day −1	Day 0	Day 1	Day 2	Day 3
**1st Experiment|Intensity of EA**					
Cisplatin, i.p.	−	+	−	−	−
EA (low intensity stimulation)	−	−	+	+	+
EA (high intensity stimulation)	−	−	+	+	+
Body weight and food intake	+	+	+	+	+
**1st Experiment|Frequency of EA**					
Cisplatin, i.p.	−	+	−	−	−
EA (10 Hz)	−	−	+	+	+
EA (100 Hz)	−	−	+	+	+
Body weight and food intake	+	+	+	+	+
**2nd Experiment|Pharmacological effects of the optimal EA conditions**					
Cisplatin, i.p.	−	+	−	−	−
EA at non-acupoint	−	−	+	+	+
EA at CV12 (low intensity stimulation, 10 Hz)	−	−	+	+	+
Body weight and food intake	+	+	+	+	+
Blood, duodenum, and brain stem	−	−	−	−	+

**Table 2:** Primers used for RT-qPCR. GHRL— ghrelin; POMC—pro-opiomelanocortin; NPY: neuropeptide Y.

Gene	Forward (5′ → 3′)	Reverse (5′ → 3′)
GHRL	AGCCCAGCAGAGAAAGGAAT	GTGGCTGCAGTTTAGCTGGT
NPY	TGTCTCAGGGCTGGATCTCT	TACTCCGCTCTGCGACACTA
POMC	GCTTCATGACCTCCGAGAAG	TCTTGATGATGGCGTTCTTG
β-actin	AAGTCCCTCACCCTCCCAAAAG	AAGCAATGCTGTCACCTTCCC

## Abbreviations

5-HIAA	5-hydroxyindoleacetic acid
5-HT	5-hydroxytryptamine
CCK	cholecystokinin
DA	dopamine
EA	electro-acupuncture
ELISA	enzyme-linked immunosorbent assay
GHRL	ghrelin
HPLC-ECD	high performance liquid chromatography-electrochemical detection
i.p.	intraperitoneal
NE	norepinephrine
NPY	neuropeptide Y
NTS	nucleus tractus solitarii
POMC	pro-opiomelanocortin
qRT-PCR	quantitative reverse transcription - polymerase chain reaction
